# The New York Institute of Stomatology

**Published:** 1899-05

**Authors:** 

**Affiliations:** The New York Institute of Stomatology


### Reports of Society Meetings.

### THE NEW YORK INSTITUTE OF STOMATOLOGY.

A REGULAR meeting of the Institute was held on Tuesday evening, February 7, 1899, at the office of Dr. Edwin S. Robinson, 28 West Thirty-ninth Street, the President, Dr. E. A. Bogue, in the chair.

The minutes of the previous meeting were read and approved.

Dr. J. Adams Bishop read extracts from the *Congressional Record* concerning the appointment of dentists to regular army service.

Dr. A. H. Brockway called attention to the publication, in an issue of the Brooklyn *Daily Eagle,* of the paper recently read before a medical society by Dr. Tuthill on "The Deleterious Effects of Mercury from Amalgam Fillings."

### COMMUNICATIONS ON THEORY AND PRACTICE.

*Dr. C. O. Kimball.*—In line of Dr. Payne's communication last month I wish to report a case that has interested me very much. It is not often that a man cares to spread his mistakes before the profession, and yet that is what I am now about to do.

A man of about forty-nine years of age, in pretty good general health, had been for several weeks past under a severe strain of anxiety and overwork. During a long railroad journey he began to have pain of a neuralgic character in his left upper bicuspids, which had gradually but steadily increased for about four or five days. He called to see me to have relief from the pain, which was becoming so severe as to interfere with his work. The pain extended over the side of the head and face, but concentrated itself especially in these teeth. I found both bicuspids had been filled with large gold stoppings, including the fissure in each tooth and covering most of the approximal surface. These had been patched with amalgam. Above these fillings each tooth was decayed, the first bicuspid decidedly, the second very slightly, and each of these cavities was somewhat sensitive. Believing at first that it was simply irritation of the dentine, I put in a little temporary gutta-percha and a slight wedge, expecting that filling the cavity would make it all right. Late that evening I received word that the pain in the teeth had increased to a very marked degree. Circumstances made it impossible for me to see him until eleven o'clock the next day. I found that the pain had increased to such an extent that he had already seen a physician, thinking possibly the trouble might arise from some other cause. His physician administered phenacetin and sent him again to me, believing the pain to arise from one of these teeth.

I found the condition of things unchanged except for the worse. The teeth were tested with hot water, which increased the pain decidedly, and with ice, which had little effect. The second bicuspid was slightly sensitive to percussion. The first not at all so. In fact, all the symptoms pointed to the second rather than to the first bicuspid, and although some were obscure, yet upon the whole it seemed to me that a clear case of pulpitis was made out, probably in the second bicuspid, though of this I was not absolutely sure. I drilled into the second bicuspid and found the pulp apparently normal; injected a little cocaine and removed the pulp at once, with but trifling pain.

The root was then thoroughly cleansed and sterilized with formaldehyde and bichloride and sealed up for the irritation of the work to subside. About three hours later I saw him again; the pain was as severe as ever and pointing just as clearly now to the first bicuspid, which, however, was not sensitive to percussion.

I explained matters fully to the patient, feeling not sure now that the tooth had anything to do with it, but after weighing the matter carefully it was decided best to devitalize the tooth. I found in this tooth a different state of things, the root being almost entirely filled with secondary dentine, so that it was not easy to trace the canal.

The pulp was removed, and this seemed to give some relief, but not as completely as we had expected.

The patient was seen the next morning. The pain in the teeth was distinctly better, but an eruption had broken out on that side of the head and face, while the corresponding half of the lips and tongue were all covered with small ulcerations.

I did not believe now that the trouble could come from the teeth alone. In the course of the day I talked over the case with his surgeon. He had seen the patient, but was still somewhat uncertain of the diagnosis, although it seemed impossible that so much disturbance to the trophic nerves could have come from reflex action. Later in the day, after consultation with another physician, it was pronounced to be an obscure case of shingles.

I have no apology to make for my action in devitalizing these two teeth. So far as I recall in my experience I have hardly ever opened a tooth of that kind and found it alive, though as a mere matter of chance anybody might make the mistake. The pain was so marked and so persistent, and simulated so exactly a slight pulpitis that had not gone far enough to extend outside of the foramen and involve the tissues beyond the tooth, that I was deceived. Though after I had opened the first one I was very doubtful about the second. I laid the matter fully before my patient, stating that I did not think there was anything wrong with that tooth, but I felt justified in opening it, because there was a bare possibility of gaining relief, and he had suffered intensely two or three days. From the time the eruption appeared the pain ceased almost entirely.

There were two other things in my mind. One was the antrum and the other was malignant disease; as we know, the presence of malignant disease in the deep tissues of the face sometimes produces intense neuralgic pain, but that, of course, I had no means of verifying until the eruption came out.

*Dr. S. H. McNaughton.*—Fifteen years ago I had herpes zoster, the eruption being in the left supraorbital and frontal region, corresponding to the distribution of the frontal branch of the first division of the fifth nerve.

For a day or two there had been an uneasy pricking sensation in the region referred to; this was followed by some swelling, pain, and eruption, and was immediately diagnosed as herpes.

The swelling increased, as did also the eruption and neuralgia.

The eye was swollen, closed, and very painful; the upper teeth on that side became tender to touch and felt elongated.

The pain simulated pericementitis rather than pulpitis, and very likely might have been mistaken for it had the teeth become tender to touch before the eruption appeared.

*The President.*—There is another condition which Dr. Kimball could not very well touch upon in this connection. I beg leave to draw your attention to it,—namely, the pain, in any one of the sinuses, frontal, ethmoidal, or antral, which often comes to people suffering from a cold, and which they cannot differentiate from pain in the teeth.

We will now have the pleasure of listening to Dr. Benjamin Lord, who has consented to tell us what materials he uses in combination for filling teeth.

*Dr. Benjamin Lord.*—Mr. President, I will present a few thoughts on the subject of the evening, "Combination Fillings," as in my experience there is very great interest and value in combining some of the materials that we use in the operation.

First, I will speak of combining gold- and tin-foils. I am accustomed to make this combination by folding the two foils in such a way that the tin will be equally distributed throughout the gold. To do this I fold a quarter of a sheet of soft gold over twice, which gives four layers or folds. I then place on the folded gold a strip of tin-foil of the same length as the gold but somewhat narrower, so that the proportion of tin shall be about one-sixth of the whole. I then fold the gold over the tin a sufficient number of times to have the strip the width required. For small cavities I would use a sixth of a sheet of gold and, consequently, less tin, and fold the two metals together in the same way. By this combination I contend that we get greater softness, toughness, more certainty in the welding, and, when properly packed and condensed with suitable instruments, greater solidity; and as a consequence, the margins are stronger. Such fillings will wear longer and preserve the teeth, or rather prevent further decay, with more certainty than those made all of gold. An amalgamation of the two metals takes place at some period after their introduction.

I consider this combination very suitable for the labial surfaces of the front teeth, though with a less proportion of tin, say about one-tenth, as the color is less conspicuous than gold. Too much cannot be said in favor of the combination of tin and gold, in proper proportions, and I believe that a thorough trial will be convincing as to the correctness of my views on this subject.

There is another combination of metals that I have used with a good deal of interest and satisfaction, and, I trust, with increased usefulness, for some two years. This consists in adding gold to the amalgam alloy in sufficient amount to make the mass contain about twenty per cent. of gold.

I use the crystal gold strips number one, prepared by A. J. Watts; as it is very spongy, it readily takes up the mercury. I add the gold to the alloy and mercury, then grind them as if no gold was being used, and a perfect amalgamation is the result.

The question at once arises, What is gained by this combination? The color is very greatly improved; I have not observed any shrinkage, expansion, or crumbling of the margins; also there is less staining of the walls of the cavity; so I consider much is gained by the addition of the gold.

*The President.*—Our distinguished guest, Dr. C. W. Strang, of Bridgeport, Conn., will now instruct us regarding the use of his particular combination of amalgam and zinc phosphate.

*Dr. C. W. Strang.*—Mr. President and gentlemen, it affords me much pleasure to be with you this evening, to address you upon a subject in which I have been very much interested for a period extending over nearly ten years of my practice. I have had the privilege of presenting the matter to a number of associations and at a number of meetings, and I also take occasion to say that the idea of combining amalgam and oxyphosphate of zinc is not original with me. About ten years ago, in one of our dental journals,—I do not remember the journal nor the name of the writer,—I saw the suggestion of combining amalgam and oxyphosphate of zinc. No description was given as to the method. I was sceptical regarding the advantages to be obtained by this combination, but I began to experiment, and found that in proper proportions and properly manipulated I was able to make a preparation that was reasonably hard, a preparation that apparently did not shrink or contract in the least; that was not appreciably affected by the secretions of the oral cavity, and that was not affected materially by weak alkalies, though this mixture when thrown into an ammonia bottle entirely disintegrated in a few hours. I found that it made but very little difference what amalgam was used, or what oxyphosphate of zinc, so long as the latter was of good quality. The use of different amalgams I found caused a variation in color of the mixture, also in the grain or homogeneousness. In my experiments Lawrence's amalgam gave as hard a mixture as any. The standard amalgam manufactured by Axfeldt & Dubois, of Philadelphia, gives a reasonably hard preparation, but very dark. The amalgam of Fox & Gerhardt gives quite a light mixture. The Fellowship alloy gives us the whitest preparation of any amalgam that I have used. The method that I have adopted in the preparation of this combination for filling-material is about as follows: If I am to insert a filling in the anterior teeth I try to get a preparation of as light a color as possible. I select Fellowship alloy, add sufficient mercury to make it a little more plastic than I would were I to use amalgam alone. I wash it with alcohol and grind the mass in a mortar. For the quantity of filings that I have used in the amalgam I take from one-third to one-half of the whitest zinc oxide I can obtain, place upon the glass tablet about the quantity of the liquid that would be needed for the amount of oxide, possibly a very little more, then grind the oxide powder with the amalgam, in a mortar, thoroughly. With a very strong spatula I now mix the phosphoric acid with the mass above described, and get it into place as speedily as possible. The material is very sticky, and considerable force should be used in packing it into the cavity. Under no circumstances should it be used unless all moisture can be excluded. I will say that I frequently see operations that I made nine years ago with this material that are as perfect as though made less than nine days ago. I remember the first case in which I used this material. The patient was about sixteen years of age, a young lady. The second superior bicuspid had erupted within the arch, so that the first bicuspid, the second bicuspid, and the first molar made a triangle. The posterior part of the first bicuspids were very badly decayed, the decay extending above the margin of the gum. The second bicuspids were decayed both anteriorly and posteriorly. The first molars were badly decayed anteriorly. I extracted the second bicuspids, which left the cavities in the molars and remaining bicuspids free. I was in doubt as to whether the first bicuspids would tolerate any filling, because the cavities were so deep, but I took the risk of using this combination. It was not necessary for me to cut away the masticating surfaces of the bicuspids, though the cavities came dangerously near them. The decay was removed, the margins of the cavities were perfectly finished, prepared as carefully as though I was preparing for gold fillings, and this combination was introduced. I had the pleasure of examining that work only a few weeks ago, and the teeth were as clear and the fillings as perfect at the margins as though they had been in but a few weeks. Possibly there is not quite the same contour as at the time the operation was performed. Now, I will say in regard to this filling that it never discolors tooth-structure. I believe it is the most perfect protection to tooth-structure of any preparation that we can introduce into the cavity. It adheres as closely to the cavity wall as the enamel adheres to the dentine. I use it in immature and poorly developed permanent teeth, and in patching gold fillings that are defective. I never find the teeth discolored as we find them discolored where we patch gold fillings with amalgam.

There is a class of cavities which for a great many years I have regarded with horror, but I do not now feel much disturbed when that class of cavities present. The cavities referred to are those located on the lingual surfaces of inferior molars. These are difficult cavities to manage, particularly when the patient is forty to forty-five years of age. It is exceedingly difficult to prepare these cavities so that amalgam, or any filling that does not closely adhere to the tooth-structure, will be retained. There is also another class of cavities that are rather troublesome,—those located on the buccal surfaces of second molars extending around posteriorly in which perhaps the whole buccal wall has softened and it becomes necessary to protect the whole surface of the tooth. I find that with this combination of amalgam and oxyphosphate of zinc these cavities are filled with more certainty of the teeth being preserved permanently than with any other preparation I have ever used. So far as the preparation of cavities for filling is concerned it does not make much difference whether we cut retaining grooves in the teeth or not, provided we can get a dam on and exclude moisture. If our material is properly manipulated we will be able to insert a filling that will be a perfect protection to the tooth.

In mixing the phosphoric acid with the mass I gradually draw the mixture together and keep adding the powder and amalgam until I get a mixture that is like a thick putty, which I then take between the thumb and finger. Everything must be ready to introduce the filling when it is mixed, and it should be placed into the cavity quickly, pressure being brought to bear upon it from all sides. If it is a proximal cavity, I use a ribbon saw between the teeth, so that the pressure may be brought in all directions upon the filling, which seems to give a finer-grained material than can be obtained without the pressure. I may be regarded by some of you as quite enthusiastic over this preparation, but my enthusiasm is a healthful one, which has come to me by a number of years' observation of the results obtained from its use. I think that my patients have been benefited by my experience and my work along that line. I have far greater confidence in fillings of this kind, particularly in soft teeth, than I have in any material I have ever used. A seam will never be found at the margin of these fillings.

In five minutes after the filling has been introduced disks and burs may be used to dress it down, but it does not get hard enough to have much force brought upon the proximal surfaces in less than ten minutes. The Fellowship alloy sets rapidly in these fillings.

Dr. Gaylord, of New Haven, used amalgam filings in the oxyphosphate without mercury, but he discontinued it because the filling was rough. Some good operators add pure amalgam to the surface of these fillings, but I would prefer to make the whole filling of the combination of amalgam and oxyphosphate, because with them there would be no danger of checking at the margins. In mixing the amalgam I am careful not to get an excess of mercury, adding more filings instead of squeezing out the mercury.

*The President.*—We will now hear from Dr. C. B. Parker, who will kindly favor us with his method of combining alloy filings with oxyphosphate of zinc.

*Dr. C. B. Parker.*—The material to be used for the saving of children's teeth, as well as the bicuspids (particularly of the adult), in our daily practice was a source of some little anxiety to me when I commenced to practise here eighteen years ago. Dr. Jarvie, with whom I was associated, usually placed the children in my care. Our practice was to use tin wherever it was practicable, where that could not be used, the next best material that was adapted to the case was resorted to.

In those sensitive and shallow cavities that were impossible to properly excavate the cements were nicely adapted, but so liable to wash out in such a short time that I began to look for some remedy. I commenced by combining the cement with amalgam, but it was never satisfactory. I found that after the alloy had been amalgamated the amalgam and cement could not be thoroughly mixed; the cement would be in one part of the filling, the amalgam in the other; so that was discarded. I next tried mixing alloy filings with the dry powder; the filling was then prepared in the same manner as ordinary cement.

I think it is particularly well adapted for children's teeth, especially on the grinding and buccal surfaces, for two reasons,—first, it is practically non-conductable; secondly, it will resist attrition in the average mouth from two to three times as long as the same cement without the combination of the alloy. I say practically non-conductable, for neither the cement nor the metal are continuous. It resists attrition, for it always presents a metallic surface. This is well illustrated in the specimens which I pass around. I wish to call your attention to the difference in the fillings where pressure was used in one and not in the other, as is the condition in all plastic fillings where not enough pressure is exerted until crystallization commences or is fairly under way.

In a case where I dare not use much force I partly fill the cavity as gently as possible, after allowing it to harden a minute or two, and then put in the remainder of the filling, keeping it under gentle pressure until it has hardened sufficiently. By so doing the atoms are held in such close contact that the lasting and beneficial qualities have been improved in the same ratio over a filling in which no pressure had been used.

Another class of teeth where we have considerable annoyance are the bicuspids when they are so broken down that we are not justified in filling them with gold and the use of amalgam would produce so much discoloration as to be objectionable. In these cases we resort to cement or gutta-percha. In a few months the patient returns saying that the fillings are so worn down that he is very much annoyed by food always lodging there.

In nearly all such cases it will be safe to use this material, with the full knowledge that there will not be the slightest discoloration, any more than if the cement alone was used, especially if pressure on the filling has been continued until crystallization was well advanced, for the cement will always appear on the outer surface, and the filling will give better service than any cement I ever saw.

Within the past month I have seen two patients who had molars and bicuspids filled with this material by two of our New York members at about the time that I read a short paper on this subject before the Odontological Society in October, 1889. The fillings are still in a good state of preservation. I very often see fillings that I put in from six to ten years ago.

The preparation of this filling-material can be easily done by any one. I find that with the average oxyphosphate powder equal parts of the alloy filings should be added. And right here let me say not to use a coarse-cut alloy; it must be a medium or medium to fine cut, as the coarse shaving class of filings will flake off from the surface.

Place the combined materials in a bottle, shake and rotate until they are thoroughly mixed, and then prepare in the usual way for filling by mixing with the phosphoric acid.

In selecting the oxyphosphate I use the light color. In fact, for fillings in the anterior teeth I use the white oxyphosphate, three parts, to alloy, two parts. The combination of the alloy with the oxyphosphate delays the crystallization a little, and I should always advise the application of a varnish, or any other suitable substance, to the surface of the filling to prevent contact with water.

Now, after fifteen years' experience with this material in the mouth of the young child, as well as the adult, I firmly believe that no mistake will be made in using this combination wherever cement would be used, with the full knowledge that in so doing the lasting qualities of the filling will be very much improved.

*The President.*—Perhaps Dr. Parker can tell us if he has tried using gold filings to make combination fillings.

*Dr. Parker.*—I have used the gold filings, but that makes a rough filling; when the cement dissolves it leaves a rough surface. I do not think there is any advantage in the gold.

*Dr. Lord.*—I simply wish to remark that I would prefer tin-foil in the grinding surfaces of children's teeth, either for the deciduous or permanent, and also for the proximal sides if the cavities are small. The trouble is that we do not have the children brought to us generally until the teeth are very much broken down. In such cases I fill cavities and spaces, for greater comfort, using gutta-percha at the cervical margins and covering this with oxyphosphate, as it wears longer.

When tin is used to preserve the teeth until they become fully developed it is not necessary to keep the work dry. This is a great relief to children, and the more simply young persons' teeth can be treated the better, so long as we secure the end in view.

*Dr. J. Morgan Howe.*—I have been especially interested in the description of Dr. Strang's method of combining amalgam and zinc phosphate, which is new to me. I think it must be destined to be of great service to us. I have been in the habit of using the combination of alloy cuttings with the phosphate cement that Dr. Parker suggested and introduced some ten years ago ever since that time, and have found it to be a great improvement in wearing quality over the cement alone. I have been annoyed in some cases by the filling assuming a rough surface, and I think he has explained the reason. I have attributed it to the wasting of the cement, leaving the metal points prominent. I suppose that by using finer particles of metal the results would have been more satisfactory in that respect. Whether the combination of alloy filings with cement or amalgam with cement is going to prove the better will be for us to determine by experience. The combination of tin and gold I have recently used very little. My experience with it has not been entirely satisfactory, and I have not used it at all, I think, for three or four years. The other combination of gold with amalgam that Dr. Lord spoke of, I have not used at all in the way he suggests.

*Dr. Louis Shaw.*—I do not see the advantage of alloy filings over any other insoluble substance, such as powdered silica. If there is any advantage I would be glad to know of it.

*Dr. Howe.*—From my observation I should say that there is a decided difference between a mixture of alloy filings with phosphate cement and a similar mixture of such substances as broken-up teeth or filings of gold, on which the phosphoric acid would have no chemical action. One reason for the good results obtained by mixing particles of silver-tin alloy with cement is that the metallic pieces are held in the combination by chemical action on their surfaces.

*Dr. McNaughton.*—I have here an upper wisdom-tooth which I shall pass around. In it there are two fillings of gold and tin put in by one of our best operators. These fillings were in the mouth for about nine years. The filling in the distal surface must have been an exceedingly difficult one to put in, as it was almost an impossibility to see the cavity, which was close to or above the gum margin, and there was very little if any undercut. This filling is much disintegrated and soft. The dentist who inserted it thought that moisture must have entered during the operation. The filling in the mesial surface is clean and bright, and in every way a perfect filling. It is in contact with a gold filling. These two fillings prove the saving qualities of the combination, and show how differently the same materials may act as regards disintegration.

*Dr. Kimball.*—I have received the following letter from Dr. H. W. Gillett, of Newport:

"Newport, R. I., February 4, 1899.

"My dear Doctor,—Apropos of Tuesday's discussion by the Institute, the following may possibly be of interest.

"In April, 1889, a case presented with defective amalgam fillings in right superior first molar and second bicuspid, corono-approximal.

"The teeth had been much cut away previously, and the flat fillings had allowed them to approach each other, closing the space.

"They were wedged apart first.

"When prepared, the molar cavity was large and extended above the gum, while the bicuspid cavity was one of those distressing saucer-shaped cavities reaching far above the gum, with which we are all familiar as the sequence of so-called permanent separation of bicuspids.

"These were filled by what many of us know as the Clapp method,—matrix; amalgam (probably Eckfelt & DuBois standard) for first third or half of cavity; a crystal gold packed into this at once till mercury failed to show any more, and finished with foil.

"Dr. A. R. Eaton, of Elizabeth, N. J., in whose care the patient now is, told me last month that those two fillings are still in good condition.

"These are among the first that I did in that way of which I know the subsequent history.

"The cavity was prepared with undercuts as for amalgam or gold, and the fillings were retained without any assistance from cement, as has been later recommended by Dr. Clapp.

"I continue to use the method with satisfaction in selected cases.

"Yours very truly,

"Henry W. Gillett."

*Dr. J. Bond Littig.*—Dr. Parker in his paper has fully described my experience with this combination filling. I have used it ever since, and, in fact, previous to, the time Dr. Parker read his paper before the Odontological Society, having heard through others that he was using such a filling.

I find it one of the most valuable materials for the temporary teeth, and occasionally use it for the permanent ones. I have had the pleasure of seeing some of these fillings put in, I think, by Dr. Parker some eight years ago on the grinding surfaces of lower molar teeth of an adult, and they were in as perfect condition as the day they were inserted.

In using this filling for adults I find that it wears more rapidly in some mouths than in others, resembling in some respects the oxyphosphate, but superior to it as a filling for temporary teeth. There is a chemical affinity between the phosphoric acid and the metal, therefore I generally mix them together and then stir in the powder until I get the proper consistency.

The reason this filling gets rough is because the filings used are too coarse. If a filling should become rough, a burnisher rubbed over it a very few times will restore the surface.

*The President.*—Dr. Watkins, have you had any experience in combination fillings?

*Dr. S. C. G. Watkins.*—I have heard so many interesting and new things this evening that I feel at a loss for anything to say myself. Dr. Lord's method of mixing tin and gold I was very much interested in. I have used them separately, partly filling the cavity with tin and then adding the gold, but I will try Dr. Lord's method, as I believe it to be a good one. In regard to using amalgam according to the methods described here to-night, I have had very little experience, and that was not satisfactory. From what I heard this evening I presume it is my own fault that I have not had better results. I shall also experiment in this line. The method which I have been using in combination fillings to quite an extent in large cavities and fragile teeth, has been to first partly fill the cavity with soft oxyphosphate, and then press gold or amalgam directly into it, waiting for the cement to set; the edges would then be cleaned and made free from cement, and the gold or amalgam built up until the filling was completed. I presume that is known as Dr. Clapp's method. I do not know whether that is really Dr. Clapp's original idea or not, because it has been used a great many years. I know I used the oxyphosphate and gold about eighteen years ago, and I have one particular filling put in in that way, in 1883, which I am very proud of. I use that method at the present time, and I feel well satisfied with it. The mixing of amalgam and gold, by partly filling a cavity with amalgam and then adding gold to that, I used for some years, but abandoned the method several years since. I shall certainly try the three methods which have been suggested.

*Dr. F. Milton Smith.*—It has given me great pleasure to hear Dr. Strang to-night. Some one has said, "Their works do follow them." In his case his work has preceded him, for it has been my good fortune to have seen some of his work in gold fillings which is certainly a credit to him.

On former occasions I have been a little suspicious when I have heard plastic fillings of various kinds highly commended, and have been uncharitable enough to suspect that the one endorsing them could not make good gold fillings.

Unless I am mistaken I have lately read in one of our journals an article by Dr. Strang, to which I gave only slight heed, because of not being impressed with the name of the writer. It did not occur to me that he was the same whose superior gold fillings I had seen.

Since recognizing Dr. Strang and hearing from him of his success, I am strongly impressed with the value of his combination. Since he has done the experimenting for ten years past, it seems only necessary for me to learn from him just how he mixes his filling in order to adopt the method, which I am satisfied is a good one. I feel very grateful to him for bringing it forward.

I have no question of the utility of the combinations suggested by the other gentlemen, and am sure I shall find a place for them in my practice.

It would seem to me that our Executive Committee is entitled to especial commendation for this very practical meeting.

*Dr. S. E. Davenport.*—I feel that a little more emphasis should be given to what Dr. Lord has said concerning the use of gold and tin, for the combination has seemed to me for years to be a most valuable one, especially in the crowns of the temporary teeth, and also in the crowns, buccal surfaces, and small proximal cavities of permanent teeth. When Dr. Lord referred to the change which comes to tin and gold fillings in nearly all mouths a little while after they are made, he called it amalgamation. I am at a loss to know what it ought to be called, but think that term misleading, as it implies the presence of mercury when used to define chemical change in metals.

Professor Miller, of Berlin, who used tin and gold freely and successfully years ago, told me once that he did not know what that change was. The fillings get very hard and the two metals appear, on the surface at least, to have more than a mechanical union.

Dr. Strang, with whom I used to play fifteen or twenty years ago in the old Connecticut Valley Dental Society, certainly knows how to make gold fillings, and his attention to this plastic combination was given before he arrived at an age when it becomes necessary for some of us to make things easy. His interest in this material came from his earnest desire to preserve the class of teeth which otherwise would certainly be sacrificed if gold alone were to be depended upon. I think Dr. Strang placed too little emphasis upon one of the greatest advantages possessed by this combination which he has done so much to develop for the profession. He did say it was not necessary to make deep undercuts, to be sure, but he did not emphasize sufficiently, to my mind, the fact that this combination can be used in saucer-shaped cavities almost without undercuts, depending upon the adherence of the material to the walls of the cavity, which must, of course, be absolutely dry. If the hour were not so late, I should be glad to report one or two cases from practice in illustration of this, but I think all who experiment with it will find that what I have said upon this point is true.

Because of the ability shown by Dr. Parker in putting together the little paper he has favored us with to-night, he will, I am sure, often be called upon by the Executive Committee. His combination of materials has done much to preserve the teeth of children, and many adult teeth of frail character which are perhaps better preserved by this combination than any other. I feel under obligation to all three gentlemen for their instruction this evening.

*Dr. Strang.*—I want to express my appreciation of the many compliments that have been paid me this evening, and should feel sorry to have any go away being deceived in any matter, and I am glad that my friend Davenport has undeceived all in regard to my age. I want to say that, in using the combination of amalgam and oxyphosphate of zinc, we cannot afford to be slovenly in any respect. Prepare the cavities as thoroughly as though for gold fillings, except that it is not necessary to make undercuts, retaining pits, or as much of a groove as would be necessary for a gold filling. Make the cavity absolutely dry before the filling is introduced, and be sure that not even an instant is lost, after the mass is of the proper consistency, in getting it into the cavity under pressure. I think the results then will not be disappointing. I thank you, gentlemen.

*Dr. Kimball.*—I move a vote of thanks to Dr. Strang for his most interesting and able paper.

Carried.

Adjourned.

FRED. L. BOGUE, M.D., D.D.S.,
*Editor The New York Institute of Stomatology.*

